# Proteome Expression and Survival Strategies of a Proteorhodopsin-Containing *Vibrio* Strain under Carbon and Nitrogen Limitation

**DOI:** 10.1128/msystems.01263-21

**Published:** 2022-04-06

**Authors:** Gwendolyn E. Gallagher, Jacob R. Waldbauer

**Affiliations:** a Department of the Geophysical Sciences, University of Chicago, Chicago, Illinois, USA; Los Alamos National Laboratory

**Keywords:** metabolism, nutrient limitation, photoheterotrophy, proteomics, proteorhodopsin

## Abstract

Photoheterotrophy is a widespread mode of microbial metabolism, notably in the oligotrophic surface ocean, where microbes experience chronic nutrient limitation. One especially widespread form of photoheterotrophy is based on proteorhodopsin (PR), which uses light to generate proton motive force that can drive ATP synthesis, flagellar movement, or nutrient uptake. To clarify the physiological benefits conferred by PR under nutrient stress conditions, we quantified protein-level gene expression of Vibrio campbellii CAIM 519 under both carbon and nitrogen limitation and under both light and dark conditions. Using a novel membrane proteomics strategy, we determined that PR expression is higher under C limitation than N limitation but is not light regulated. Despite expression of PR photosystems, *V. campbellii* does not exhibit any growth or survival advantages in the light and only a few proteins show significant expression differences between light and dark conditions. While protein-level proteorhodopsin expression in *V. campbellii* is clearly responsive to nutrient limitation, photoheterotrophy does not appear to play a central role in the survival physiology of this organism under these nutrient stress conditions. C limitation and N limitation, however, result in very different survival responses: under N-limited conditions, viability declines, cultivability is lost rapidly, central carbon flux through the Entner-Doudoroff pathway is increased, and ammonium is assimilated via the GS-GOGAT pathway. In contrast, C limitation drives cell dwarfing with maintenance of viability, as well as utilization of the glyoxylate shunt, glutamate dehydrogenase and anaplerotic C fixation, and a stringent response mediated by the Pho regulon.

**IMPORTANCE** Understanding the nutrient stress responses of proteorhodopsin-bearing microbes like Vibrio campbellii yields insights into microbial contributions to nutrient cycling, lifestyles of emerging pathogens in aquatic environments, and protein-level adaptations implemented during times of nutrient limitation. In addition to its broad taxonomic and geographic prevalence, the physiological role of PR is diverse, so we developed a novel proteomics strategy to quantify its expression at the protein level. We found that proteorhodopsin expression levels in this wild-type photoheterotroph under these experimental conditions, while higher under C than under N limitation, do not afford measurable light-driven growth or survival advantages. Additionally, this work links differential protein expression patterns between C- and N-limited cultures to divergent stationary-phase survival phenotypes.

## INTRODUCTION

In many natural environments, microbes experience extended periods of nutrient limitation. Cells can enter phases of slow growth or dormancy to survive until more clement conditions return, but they can also adapt evolutionarily via horizontal gene transfer to better cope with nutrient stress (1). (Meta)genomic surveys have indicated that many heterotrophic microbes living in sunlit aquatic habitats have acquired genes that enable light-driven energy metabolism, possibly in order to supplement respiration during times of carbon scarcity (2–4). To date, however, we know relatively little of how these putative photoheterotrophs regulate expression of light-driven metabolic processes in response to nutrient limitation and what the physiological impact of that expression is. These knowledge gaps limit our ability to gauge and model the ecological and evolutionary impact of these apparently widespread photoheterotrophic metabolisms.

One especially prevalent form of photoheterotrophy is based on proteorhodopsin (PR), a light-driven transmembrane proton pump that generates proton motive force that can drive ATP synthesis, flagellar movement, or nutrient uptake (5–7). The broad taxonomic and geographic distributions of PR suggest that it is an ecologically important form of photoheterotrophy. Diverse members of the phyla *Proteobacteria*, *Bacteroidetes*, and *Euryarchaeota* carry PR genes, and PR is thought to be the most abundant rhodopsin in nature (8, 9). PR has been found in the widely abundant SAR11 (*Pelagibacter*) clade of alphaproteobacteria and is estimated to be present in the genomes of 50% of photic zone bacteria in the western Sargasso Sea (10) and 13% in the Mediterranean Sea (11). Proteorhodopsins have recently been suggested to absorb as much light energy as chlorophyll *a* in some marine habitats (12).

Although PR is widely distributed, our understanding of its functional role in the physiologies of the diversity of microbes that carry it is far from clear. One prevailing hypothesis is that generating ATP via PR may supplement heterotrophs’ energy metabolism during carbon starvation periods (2, 3). Perhaps the most specific experimental evidence in support of this idea has been seen in Vibrio campbellii BAA-1116, which has a survival advantage in the light (under azide-induced respiratory inhibition) that has been linked to PR-driven ATP production (13). “*Candidatus* Pelagibacter ubique” (2) and *Dokdonia* strain MED134 (14) show differential light/dark gene expression under carbon starvation conditions, and heterologous expression of PR in Escherichia coli alters respiration (6). Some PR-containing, nominally photoheterotrophic microbes show differential growth under light and dark conditions: *Dokdonia* strain MED134 has a growth advantage in the light (15), *Vibrio* strain AND4 has a survival advantage in the light, but light does not have any apparent effect on growth of PR-containing strain SAR11 under conditions tested to date (16–18). These results regarding PR’s contribution to growth physiology highlight the diversity in how PR expression contributes to photoheterotrophic metabolism.

One notable impediment to understanding proteorhodopsin’s physiological role is the absence of protein-level quantification of its expression, which could clarify the extent of its contribution to cellular energy budgets. At the transcript level, several PR expression patterns have been observed: a peak of PR transcription in mid-exponential phase of *Dokdonia* strain MED134 corresponded with a growth advantage in the light, while a peak of PR transcription in late exponential/early stationary phase of *Vibrio* sp. AND4 corresponded with a survival advantage in the light (16, 17). In *V. campbellii* BAA-1116, PR transcription is induced both by light and during stationary phase (13). PR protein expression was quantified in Photobacterium angustum S14 using a luciferase reporter construct, demonstrating that PR expression is responsive to light in that organism (19). To date, protein-level PR expression has been quantified only once by proteomics in a wild-type strain (20), likely due to the structure and membrane localization of proteorhodopsin, which comprises 7 transmembrane alpha-helices with only small extramembranal loops, leaving few portions of the protein readily protease accessible to generate the soluble peptides that are most detectable by liquid chromatography-mass spectrometry (LC-MS). With emerging recognition of the extent and quantitative importance of posttranscriptional regulation in bacteria (21, 22), it is uncertain how transcript-level expression patterns relate to the abundance of PR photosystems.

To explore the role of proteorhodopsin in aquatic photoheterotrophs’ response to nutrient limitation, we measured growth physiology and proteome expression of the marine gammaproteobacterium Vibrio campbellii CAIM 519 under carbon- and nitrogen-limiting conditions. This strain contains PR and a complete biosynthesis pathway for the retinal chromophore, akin to many nominally photoheterotrophic sequence assemblies seen in metagenomic data sets. We compared growth, viability and protein expression patterns—including of integral membrane proteins using a novel enrichment and isotope-labeling approach—in batch cultures from exponential growth through stationary phase under light and dark conditions. The relatively large sample requirements for membrane proteomic analysis necessitate experimental conditions that support high biomass growth. The copiotrophic, boom-and-bust lifestyle of some planktonic marine vibrios make them useful models for exploring the ecophysiological role of PR in surviving acute nutrient starvation following rapid biomass accumulation. In prior work, for example, a light-induced survival advantage was observed in stationary-phase *Vibrio* sp. AND4 cultures that achieved an optical density (OD) of 1.5 (17). In our experiments, either carbon or nitrogen limitation was imposed solely by shifting the ratio of C to N substrates in defined media. By studying both C and N limitation conditions, this work explored the relationship between specific nutrient stresses and PR expression. The data yield new insights into the light-driven metabolism and survival/dormancy responses of PR-bearing microbes under different nutrient limitation regimes.

## RESULTS AND DISCUSSION

### Growth physiology and survival under C and N limitation.

**FIG 1:**
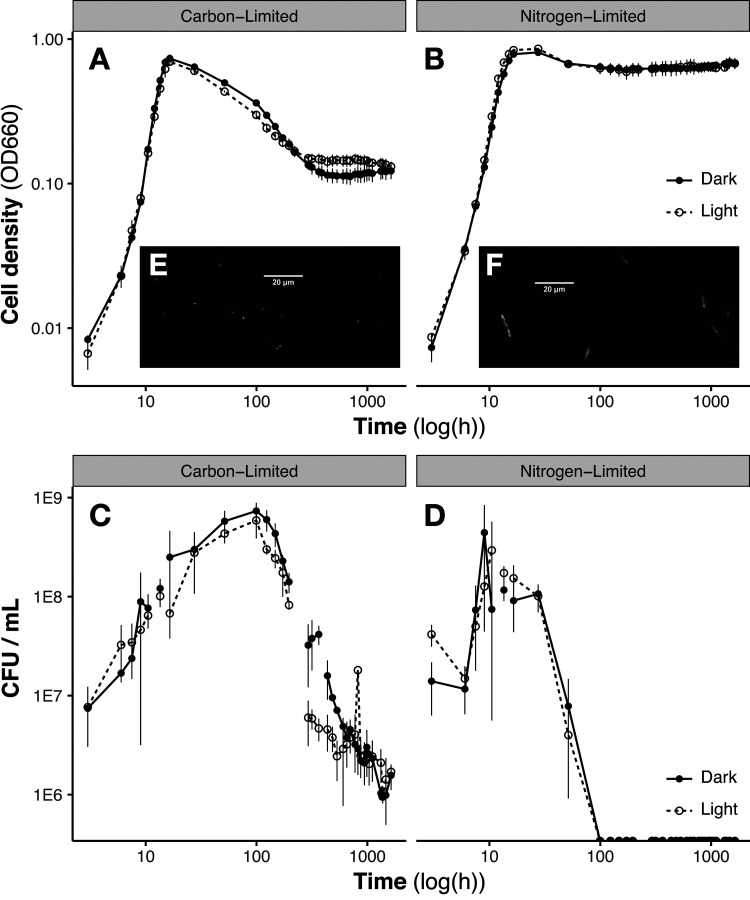
Cell growth (A and B) and CFU (C and D) for *V. campbellii* CAIM 519 in carbon-limited (A and C) and nitrogen-limited (B and D) defined media; note logarithmic axes. Filled circles/solid lines indicate the continuous dark condition, and the open circles/dashed lines indicate the continuous light condition. As the cultures enter stationary phase, optical density dramatically drops under the carbon-limited condition but cultivability is maintained. Under the nitrogen-limited condition, OD is maintained, but cells cannot be regrown after ∼100 h. No significant differences were observed between light and dark growth. (E and F) Red autofluorescence images (magnification, ×100) of stationary-phase cells under C-limiting (E) or N-limiting (F) conditions. C limitation drives cells to become smaller and coccoid, while under N limitation, cell size and rod-shaped morphology are maintained (Table S2).

*V. campbellii* CAIM 519 was grown under continuous light (300 μmol photons m^−2^ s^−1^) and dark conditions in defined media (see Table S1 in the supplemental material) where cells entered stationary phase due to either carbon or nitrogen limitation (Fig. S1). The only difference between the C- and N-limited media was the ratio of the sole carbon (maltose) and nitrogen (ammonium) sources: 17:1 C to N under the C-limited condition and 100:1 C to N under the N-limited condition. The two media supported similar maximum exponential growth rates (μ_max_ = 0.14 and 0.15 h^−1^) and growth yields (OD_max_ = 0.72 and 0.83) (Fig. 1).

10.1128/mSystems.01263-21.1 FIG S1 Growth curves (optical density at 660 nm) *V. campbellii* CAIM 519 in defined minimal media (Table S1) demonstrating carbon and nitrogen limitation. Filled circles/solid lines indicate continuous dark condition and the open circles/dashed lines indicate continuous light condition. (A) The media with differing concentrations of carbon have fixed, replete concentrations of nitrogen (2 mM NH_4_Cl). Maltose concentrations are 1.9, 2.78, and 5.56 mM. Both the cultures grown in 2.78 and 1.9 mM maltose media are carbon limited, as determined by maximum optical density. (B) The media with differing concentrations of nitrogen have fixed, replete concentrations of carbon (8.34 mM maltose). Nitrogen concentrations are 0.1, 0.2, 0.5, 1, and 2 mM NH_4_Cl. The cultures grown in 0.1, 0.2, 0.5, and 1 mM NH_4_Cl are nitrogen limited, as determined by maximum optical density. No significant differences were observed between light and dark. Download FIG S1, PDF file, 0.2 MB. Copyright © 2022 Gallagher and Waldbauer. 2022 Gallagher and Waldbauer. https://creativecommons.org/licenses/by/4.0/ This content is distributed under the terms of the Creative Commons Attribution 4.0 International license.

10.1128/mSystems.01263-21.7 TABLE S1 Composition of C- and N-limiting defined artificial seawater media. The concentrations of salts and trace metals are the same, but the C/N ratio for the C-limited medium is 17:1 and that for the N-limited medium is 100:1. Download Table S1, PDF file, 0.03 MB. Copyright © 2022 Gallagher and Waldbauer. 2022 Gallagher and Waldbauer. https://creativecommons.org/licenses/by/4.0/ This content is distributed under the terms of the Creative Commons Attribution 4.0 International license.

10.1128/mSystems.01263-21.8 TABLE S2 *V. campbellii* CAIM 519 cell sizes under different growth conditions. Download Table S2, PDF file, 0.03 MB. Copyright © 2022 Gallagher and Waldbauer. 2022 Gallagher and Waldbauer. https://creativecommons.org/licenses/by/4.0/ This content is distributed under the terms of the Creative Commons Attribution 4.0 International license.

Despite changing only the ratios of nitrogen to carbon between these two growth conditions, we observed very different survival responses in stationary phase. The optical density of the carbon-limited cultures dropped dramatically after the cells reached stationary phase, but the cells remained cultivable for at least 60 days (though with declining CFU). Under the nitrogen-limited condition, the cultures maintained optical density, but they quickly lost cultivability as the cells entered stationary phase and could not be resuscitated (Fig. 1). From 99.5 h on, multiple attempts were made to resuscitate the N-limited cultures, including plating on marine agar plates (full and half-concentration supplemented with 2 mM NH_4_Cl), as well as inoculation into multiple liquid media, including artificial seawater (ASW) with 5.56 mM maltose and 2 mM NH_4_Cl, full-strength marine broth, and 10% marine broth/90% ASW. Each attempt was done in triplicate, under both light and dark conditions. No growth, as monitored by colonies on plates or optical density at 600 nm (OD_660_), was observed in any of the media. The majority of cells (>80%) maintained membrane integrity (as assayed by LIVE/DEAD staining) into C-limited stationary phase, while, under N limitation, only a small proportion did (roughly 20% in the dark and less than 5% in the light) (Fig. S2A), indicating reduced viability under N limitation compared to that under C limitation. Cellular ATP assays (Bac-Titer Glo) showed no increase in dissolved ATP content in N-limited cultures upon heat shock lysis (unlike C-limited cultures [Fig. S2B]), which is further indication of compromised membrane integrity in N-limited cells. *V. campbellii* CAIM 519 was predominantly rod shaped during exponential growth in both media and remained so during N-limited stationary phase, but it became smaller and coccoid in C-limited stationary phase (Fig. 1E and F; Table S2). Unlike for *V. campbellii* BAA-1116 (which showed more rapid loss of cell density in the light) (13), we observed no significant differences between viability, optical density, growth rate (Fig. 1), or cell morphology (Table S2) in the light compared to the dark condition in either growth medium.

10.1128/mSystems.01263-21.2 FIG S2 (A) Viability of *V. campbellii* CAIM 519 under carbon-limited and nitrogen-limited growth conditions. Alive percentages were determined on triplicate cultures using a LIVE/DEAD BacLight bacterial viability kit (Thermo) and stained cells (∼2,000 to 6,000 per sample) were counted with a CytoFLEX S cytometer (Beckman Coulter). (B) Dissolved ATP contents (Bac-Titer Glo) of stationary-phase *V. campbellii* cultures, both pre- and post-lysis by heat shock. N-limited cultures (unlike C-limited ones) show no increase in dissolved ATP after heat shock, indicating that membrane integrity was already compromised in those cells. Download FIG S2, PDF file, 0.2 MB. Copyright © 2022 Gallagher and Waldbauer. 2022 Gallagher and Waldbauer. https://creativecommons.org/licenses/by/4.0/ This content is distributed under the terms of the Creative Commons Attribution 4.0 International license.

Analogous patterns in growth physiology and cultivability under N and C limitation have been observed in other *Vibrio* strains with and without PR: Photobacterium angustum (previously *Vibrio* sp. S14), for example, maintains CFU under C limitation but not P or N limitation (23, 24), and N-starved Vibrio vulnificus loses cultivability at cold temperatures while C- and P-starved cells maintain CFU (25). Dormancy in response to stresses (including temperature shock and antibiotic exposure as well as nutrient starvation) in diverse microbes has been characterized as a continuum of coexisting subpopulations progressing from actively growing cells through a “persister” subpopulation to a viable but nonculturable (VBNC) state (26). In this case, N starvation seems to rapidly drive *V. campbellii* into a nonculturable state (along with substantial reduction in viability); while our efforts to resuscitate the N-starved cultures were unsuccessful, resuscitation can require specific treatments distinct from alleviation of the original stressor (27). The cold shock-induced VBNC state in other *Vibrio* strains often also includes cell dwarfing (28), which we observed under C but not N limitation. Our observations of a biphasic stationary OD curve under carbon limitation is consistent with prior *Vibrio* work (23, 24) that showed that only C starvation (as opposed to P or N) induced long-term survival and cell dwarfing. While the observed cell dwarfing and maintained membrane integrity in C-starved cells are consistent with the VBNC phenotype, these cells—like C-starved V. vulnificus subjected to cold shock (25)—also remain cultivable and thus not in the VBNC state. Nitrogen starvation in *P. angustum* S14 has been shown to cause more cell death and less stress resistance than occur in cultures under C starvation (23). Our observations suggest that the dormancy response to C starvation in *V. campbellii* resembles the VBNC state in some respects (cell dwarfing and membrane integrity), yet cultivability is maintained. The decline in both viability and CFU during N starvation, on the other hand, is more suggestive of a shrinking persister-type subpopulation (26).

### Protein- and transcript-level proteorhodopsin expression.

**FIG 2:**
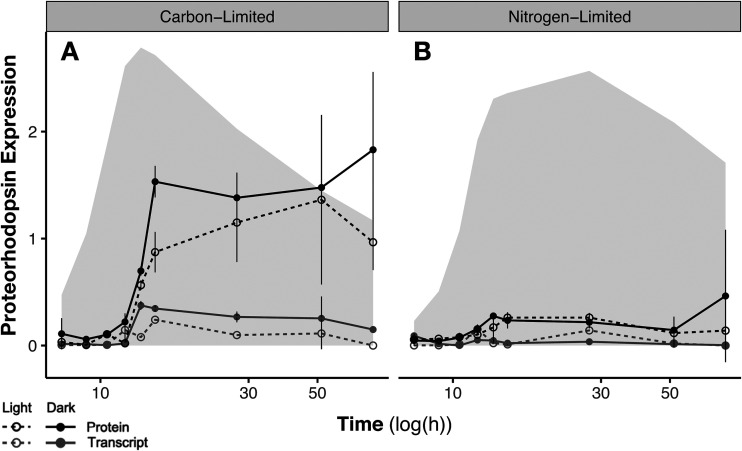
Transcript-level and protein-level proteorhodopsin (PR) expression time series for carbon-limited (A) and nitrogen-limited (B) *V. campbellii* cultures under light and dark growth conditions. The shaded region indicates growth curve (OD_660_ [Fig. 1A and B]). Transcript-level PR expression (normalized to *recA*) peaks during the transition between exponential and stationary phases under both conditions. Protein-level PR expression (quantified using two synthetic standard peptides) is higher under carbon-limited conditions and persists through stationary phase. Error bars represent combined analytical and biological variance across triplicate cultures.

We quantified proteorhodopsin expression at both the transcript and protein levels to determine how it varied with light conditions, nutrient limitation, and growth phase. At the RNA level, PR expression peaked during early stationary phase under the carbon-limited, and to a smaller extent under the nitrogen-limited, condition (Fig. 2); *Vibrio* sp. AND4 shows similar timing of PR transcription (8, 17). PR transcription was somewhat higher (2.3-fold) in the dark than the light through the transition and stationary phases under C-limiting conditions, while it was slightly higher in the light than the dark during the transitional phase of N-limited growth.

Protein-level PR expression increased over the course of the exponential phase and reached a plateau around the transition to stationary phase, where it was maintained long after the peak in transcript-level PR expression. The stationary-phase plateau in PR protein abundance was 4.4-fold higher under carbon-limiting than under nitrogen-limiting conditions. The slightly enhanced transcription of PR in the dark under C limitation was reflected in modestly elevated protein abundance during stationary phase, but no light-dark difference in protein abundance was observed during N-limited growth. In contrast, in Photobacterium angustum S14, PR protein expression is clearly regulated by blue light (19). Prior work has shown that Vibrio campbellii BAA-1116 induces PR transcription with both light and nutrient starvation and *Vibrio* sp. AND4 induces PR transcription with only nutrient limitation (13, 17). Proteorhodopsin protein expression in *V. campbellii* CAIM 519 appears to be more responsive to nutrient limitation than to light availability. On a copy-per-cell basis, proteorhodopsin reached a maximum expression level of 5,606 copies/cell in these experiments (Fig. S3). This is slightly higher than the ∼1,500 to 2,000 copies/cell inferred on the basis of retinal content for *Vibrio* sp. AND4—a strain that does have survival advantage under some light conditions (29)—but substantially lower than the up to 145,000 copies/cell inferred for planktonic cells in the Mediterranean Sea and Atlantic Ocean (12).

10.1128/mSystems.01263-21.3 FIG S3 PR copies/cell under C-limited conditions. PR copies determined by peak-area ratios with 2 synthetic, isotope-labeled peptide standards of known absolute concentration (see Materials and Methods in the main text). Cell density was determined from optical density (OD) via calibration of OD against counts of CFU per milliliter and volumetric cell counts by flow cytometry. Download FIG S3, PDF file, 0.03 MB. Copyright © 2022 Gallagher and Waldbauer. 2022 Gallagher and Waldbauer. https://creativecommons.org/licenses/by/4.0/ This content is distributed under the terms of the Creative Commons Attribution 4.0 International license.

Previous studies of Vibrio campbellii BAA-1116 and *Vibrio* sp. AND4 have linked PR transcription regulation to *rpoS*, a sigma factor that is associated with various environmental stresses, the stringent response, and induction into stationary phase (13, 17). We observed that both *rpoS* transcript and RpoS protein levels peaked during the transition to stationary phase—though the peak was much larger in mRNA than protein—irrespective of limiting nutrient or light condition (Fig. S4), and this peak corresponded temporally to the increase in PR transcription.

10.1128/mSystems.01263-21.4 FIG S4 Transcript-level (gray lines, right axis) and protein-level (black lines, left axis) *rpoS* expression time series for carbon-limited (A) and nitrogen-limited (B) *V. campbellii* CAIM 519 cultures under light (dashed lines/open symbols) and dark (solid lines/filled symbols) growth conditions. The shaded region indicates growth curve (OD_660_). Transcript-level *rpoS* expression peaks in mid-exponential phase. Protein-level RpoS (quantified by diDO-IPTL) peaks in mid-exponential phase and decreases expression levels through stationary phase. Download FIG S4, PDF file, 0.08 MB. Copyright © 2022 Gallagher and Waldbauer. 2022 Gallagher and Waldbauer. https://creativecommons.org/licenses/by/4.0/ This content is distributed under the terms of the Creative Commons Attribution 4.0 International license.

We also explored the expression patterns of genes involved in the biosynthesis of the retinal chromophore of proteorhodopsin. While no protein expression time courses of retinal biosynthesis enzymes were detected, we quantified the mRNA-level expression of *blh*, the gene encoding the dioxygenase that cleaves beta-carotene into two molecules of *trans*-retinal as the final step in the chromophore synthesis, by quantitative PCR (qPCR). *blh* showed generally consistent transcription patterns across all the conditions, with a significant initial peak during the transition to stationary phase and then a second, larger peak deeper into stationary phase (Fig. S5). Though the data are limited to mRNA abundances of one gene, these patterns suggest that retinal chromophore biosynthesis is regulated, at least transcriptionally, in a manner broadly concordant with PR.

10.1128/mSystems.01263-21.5 FIG S5 Transcript-level expression for retinal biosynthesis gene *blh* (normalized to *recA*) of carbon-limited (A) and nitrogen-limited (B) *V. campbellii* CAIM 519 cultures under light (dashed lines/open symbols) and dark (solid lines/filled symbols) growth conditions. Stars indicate significant (*P *< 0.05) local maximum peaks in the time series above mid-exponential phase expression level at *t* = 9 h. Download FIG S5, PDF file, 0.6 MB. Copyright © 2022 Gallagher and Waldbauer. 2022 Gallagher and Waldbauer. https://creativecommons.org/licenses/by/4.0/ This content is distributed under the terms of the Creative Commons Attribution 4.0 International license.

### Light-dark differences in protein expression.

While PR expression in *V. campbellii* was not strongly modulated by light, we examined the rest of the proteome for indicators of photoheterotrophy and protein-level responses to light availability. Of the 1,933 proteins in our proteomic data set, we detected only 11 with significantly differential expression between light and dark conditions: 8 proteins with higher expression under light conditions and 3 proteins with higher expression under dark conditions (Table 1). Notably, just two of these 11 proteins (ferritin and hypothetical protein 03596 [Hyp03596]) were differentially abundant between light and dark under both C- and N-limited growth; all others exhibited light/dark differences in growth on one medium but not the other.

**TABLE 1:** Proteins with significantly differential expression between the light and dark conditions^*a*^

Condition and protein	Light/dark fold change (log_2_)			
	All phases	Exponential	Transition	Stationary
Carbon limited				
Ferritin*				2.29
Hyp03596*	2.87			3.82
Hyp01996	2.01			
Methionine sulfoxide reductase A				1.36
Nitrogen limited				
Ferritin*				0.70
Hyp03596*	1.96	1.46	2.53	2.21
Hyp03791	1.76	1.94		2.74
Glutaredoxin				−1.34
Hyp19500				0.99
Hyp08520				−1.19
Azurin				1.89
ArgD				1.20
d,d-CPase				−1.57

a Positive values indicate higher expression in the light. Asterisks indicate proteins differentially expressed between light and dark in both media. “All phases” column indicates significantly different protein abundance throughout the whole time course; “Exponential,” “Transition,” and “Stationary” columns indicate during which phase(s) of the growth curve the protein expression is significantly different between light and dark conditions.

Eight proteins with differential light/dark expression are involved in reactive oxygen species (ROS) stress response, including glutaredoxin, which in Vibrio cholerae is regulated by OxyR and whose expression has been shown to increase with exposure to hydrogen peroxide (30, 31). Methionine sulfoxide reductase A (MsrA) is expressed in response to the presence of methionine sulfoxide and misfolded proteins, which can result from ROS stress (32). These two sulfur redox-active enzymes show opposite regulation with regard to both nutrient limitation and light level: glutaredoxin was more abundant in the dark than light in N-limited stationary phase, while MsrA was more abundant in the light in C-limited stationary phase, suggesting that some principal targets of ROS damage differ between C- and N-limited cells. The iron storage protein ferritin was more highly expressed in the light during stationary phase in both media. Under the Fe-replete conditions of this experiment, this expression pattern may reflect light-induced ROS stress, as cells sought to better sequester intracellular Fe to avoid the damaging effects of Fenton radical chemistry (19, 33). Ferritin expression exhibited a larger light response in the carbon-limited than in the nitrogen-limited stationary phase, perhaps due to curtailed protein production under N limitation. The other 5 ROS-related light-responsive proteins are all hypothetical proteins (Hyp01996, Hyp03596, Hyp03791, Hyp19500, and Hyp08520), whose putative involvement in ROS response is inferred principally based on their gene neighborhoods (Fig. S6); the first 4 were all more abundant in the light during one or more growth phases, while Hyp08520, like glutaredoxin, was less abundant during the dark N-limited stationary phase.

10.1128/mSystems.01263-21.6 FIG S6 Gene neighborhoods of hypothetical proteins (red) with significant, protein-level light/dark expression differences. The annotated functions of neighboring genes (blue) suggest that these proteins could have functions related to reactive oxygen species (ROS) response. (A) Hyp01996’s gene neighborhood contains proteins that have previously been shown to be differentially transcriptionally expressed under light conditions, including the SnoaL-like domain containing protein and cyclopropane-fatty-acyl-phospholipid synthase (84). Cyclopropane fatty acids (CFAs) have been shown to protect bacteria from environmental stress and are less reactive to oxidation (85, 86). (B) Hyp03596 is one of only two genes to show differential expression between light and dark conditions under both C- and N-limited growth (see Table 1 in main text); it is adjacent to RNA polymerase sigma-70 factor and ChrR genes, which comprise the ChrR-σE transcription complex and have been shown in V. cholerae to regulate the blue light-induced ROS response (84). (C) Hyp03791 is situated between a deoxyribodipyrimidine photolyase family protein and flavin reductase RutF. Similar photolyase proteins in Vibrio cholerae have been characterized as blue-light receptors but are not regulatory proteins in the light-induced ROS response (84, 87). (D) Hyp19500 is potentially a zinc metalloprotein, likely for NO resistance and detoxification, based on its similarly to a protein studied in Salmonella (88, 89). (E) Hyp08520 is adjacent to an alkyl hydroperoxide reductase/thiol specific antioxidant family protein (90). Download FIG S6, PDF file, 0.03 MB. Copyright © 2022 Gallagher and Waldbauer. 2022 Gallagher and Waldbauer. https://creativecommons.org/licenses/by/4.0/ This content is distributed under the terms of the Creative Commons Attribution 4.0 International license.

Three other proteins show differential expression in the light in the N-limited stationary phase; two (azurin and ArgD) were more abundant in the light, while one (d,d-transpeptidase) was more abundant in the dark. ArgD is part of an arginine salvage pathway that converts arginine plus 2-oxoglutarate to 2 ammonium plus 2 glutamate, enabling recycling of this nitrogen-rich amino acid. In Vibrio parahaemolyticus, this protein and the arginine biosynthesis pathway are important for survival under low-temperature conditions (34), which can induce a VBNC state, perhaps indicating a specific survival technique under light, N-limiting conditions. d,d-Transpeptidase may also be related to the noncultivable state observed under N-limited conditions, as this peptidoglycan cross-linking enzyme is important in *Vibrio* for cell morphology, growth, and homeostasis (35).

That *V. campbellii* CAIM 519 gene expression does not broadly respond to light is distinct from the case for a number of other photoheterotrophs with PR: in *V. campbellii* BAA-1116, Photobacterium angustum S14, and *Dokdonia* sp. PRO95, DSW-1 and MED134 PR expression is light responsive (19), and in MED134, retinal biosynthesis, carbon fixation, glyoxylate shunt, transporters, electron transport chain, and bacterial cryptochrome and DNA photolyase, amounting to 20% of its genome, are all regulated (at least transcriptionally) in response to light availability (7, 8, 14–16). In the SAR11 HTCC1062 strain, where PR-driven ATP production can substitute for carbon respiration during energy starvation, 10% of its transcriptome was light responsive (2, 18). Other photoheterotrophs, such as those containing genes for bacteriochlorophyll-based aerobic anoxygenic photosystems, also have broad expression changes in the light (36, 37).

### Expression of C and N metabolism during C- and N-limited growth.

**FIG 3:**
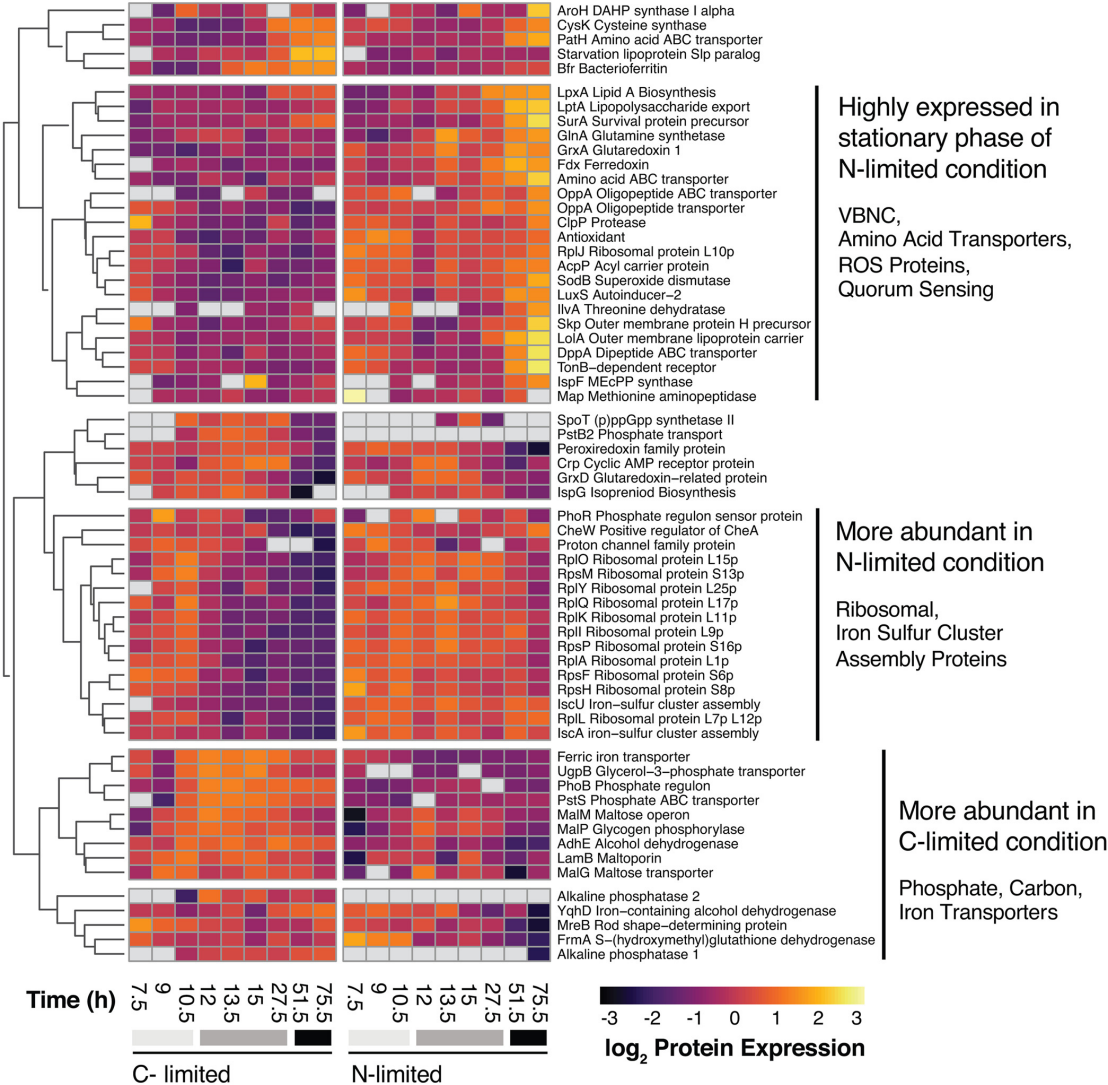
Abundance patterns of selected proteins that are differentially expressed between C- and N-limited growth in *V. campbellii*. Gray bars underneath the times correspond to observed growth phases: light gray is exponential growth, gray is the transition from exponential to stationary phase, and black is stationary phase. Proteins were clustered according to similarity in abundance time courses; prominent metabolic functions in each cluster are highlighted at the right.

While few proteins exhibited differential light/dark expression in Vibrio campbellii, despite its inferred capacity for photoheterotrophy and expression of proteorhodopsin, we observed many protein-level expression differences between N and C limitation conditions. The differential survival phenotypes seen for *V. campbellii* under C versus N limitation are associated with differential gene expression responses that include proteorhodopsin, but given the absence of a light effect on cell growth or survival, these phenotypes do not appear to be mediated by PR’s proton-pumping activity. Of the 1,933 proteins quantified, 103 showed differential expression between C and N limitation in the exponential growth phase (when data from dark and light conditions were combined [Table S3]). Sugar ABC transporter periplasmic protein UgpB, maltose transporter permease MalG, maltodextrin phosphorylase MalP, MalM, and maltoporin LamB were more highly expressed in exponential phase in the C-limited growth medium than in the N-limited medium, indicating a response to the relatively low medium C/N ratio when still growing exponentially. On the other hand, amino acid uptake and metabolism proteins, including glutamine synthetase GlnA, threonine dehydratase IlvA, methionine aminopeptidase Map, cysteine synthase A CysK, amino acid ABC transporter PatH, and dipeptide ABC transporter DppA (Fig. 3), were more highly expressed in exponential phase in N-limited media than in C-limited media, suggesting more efficient nitrogen recycling through amino acid biosynthesis and breakdown during exponential growth before N limitation is actually reached in stationary phase. The equivalent growth rates in the two media are consistent with “unrestricted growth” in the formulation of Schaechter et al. (38)—that is, limited by the type of nutrients rather than their concentration—but protein expression patterns are already “restricted” by the C/N ratio of the media.

10.1128/mSystems.01263-21.9 TABLE S3 Table of proteins with differential expression in exponential growth phase (exp), “transition” (trans), and stationary (stat) phases. The log_2_ fold change values are for the nitrogen to carbon pairwise comparisons. A positive value indicates higher relative expression under N-limited conditions, and a negative value indicated higher relative expression under C-limited conditions; “NA” indicates that there was not significant differential expression between C and N. The number of spectra quantified is included to show quality of data in the given conditions (C or N limited, light or dark, and growth phase). Also included in the table are gene name, KEGG orthology, and functional annotation. Download Table S3, CSV file, 0.4 MB. Copyright © 2022 Gallagher and Waldbauer. 2022 Gallagher and Waldbauer. https://creativecommons.org/licenses/by/4.0/ This content is distributed under the terms of the Creative Commons Attribution 4.0 International license.

Iron-containing and ROS-related proteins showed differential expression between N- and C-limited conditions, and these differences also emerged during exponential phase in some proteins. Under N limitation, iron superoxide dismutase SodB, iron-sulfur cluster assembly proteins IscU and IscA, redoxin, and two other glutaredoxins (GrxA and GrxD) were all more highly expressed starting in exponential phase. Additionally, by stationary phase, ferredoxin (Fdx) was also more highly expressed under N limitation. Aldehyde-alcohol dehydrogenase AdhE (an H_2_O_2_ scavenger [39]) was more highly expressed under C-limited conditions starting in exponential phase. By stationary phase, iron-containing alcohol dehydrogenase YqhD, bacterioferritin Bfr, ferric iron ABC transporter, *S*-(hydroxymethyl)glutathione dehydrogenase FrmA, and a glutaredoxin were all more highly expressed under C limitation as well. While previous studies have observed PR expression regulation related to iron limitation (19, 40), the iron-replete conditions in this study highlight that photoheterotrophs regulate Fe-containing proteins in response to C and N limitation as well.

**FIG 4:**
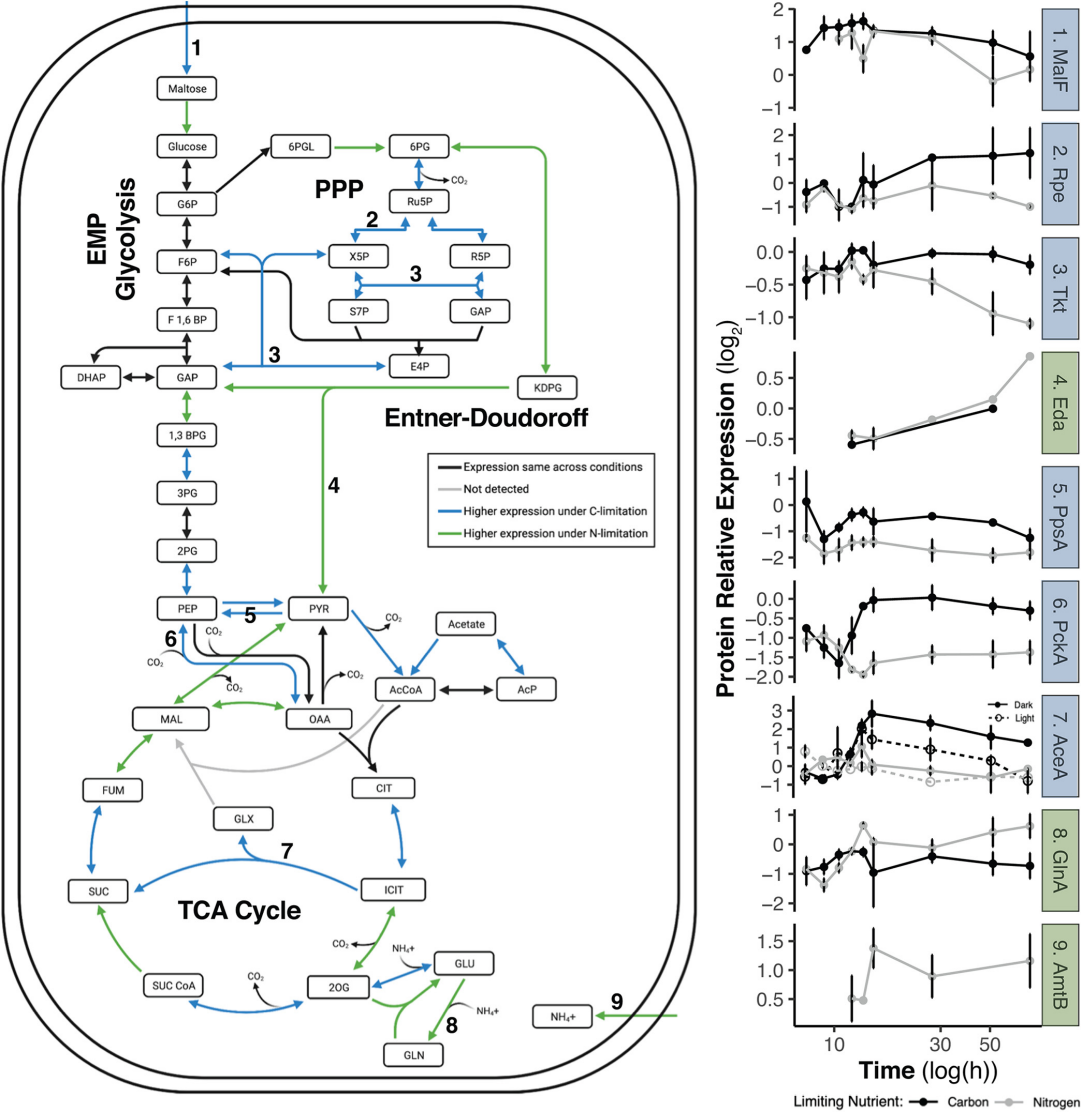
Differential expression between C- and N-limited *V. campbellii* cultures in stationary phase of key proteins involved in nutrient uptake, N assimilation, and central C metabolism. Numbered steps in pathways correspond to protein abundance time courses shown at right. C-limiting conditions result in higher expression of maltose transporters (1), pentose phosphate (2 and 3) and glyoxylate shunt (7) enzymes, and anaplerotic C fixation by PEP carboxykinase (6). N-limiting conditions drive higher expression of ammonium transport (9), Entner-Doudoroff glycolysis (4), and GS-GOGAT ammonium assimilation (8). Isocitrate lyase (7) in the glyoxylate shunt is the only depicted enzyme to show substantial light/dark expression differences, being higher in the dark under C limitation.

By stationary phase, the number of differentially expressed proteins between C- and N-limiting conditions increased to 368, notably including many enzymes involved in central carbon metabolism (Fig. 4), suggesting different strategies to maintain cellular supplies of ATP, reducing power, and biosynthetic intermediates (Table S3). In stationary phase, maltose/maltodextrin ABC transporters MalG and MalF were more highly expressed under C-limiting than under N-limiting conditions, as was the starvation lipoprotein Slp paralog protein, consistent with C starvation expression patterns seen in E. coli (41). One notable expression signal in C-limited stationary phase was increased utilization of the glyoxylate shunt via isocitrate lyase AceA, which bypasses CO_2_-losing steps in the tricarboxylic acid (TCA) cycle, thereby conserving fixed carbon. Isocitrate lyase also showed a tendency to higher expression in the dark than the light (though it did not pass our test for significantly differential light/dark expression), consistent with expression patterns found in cyanobacteria (42) but opposite to those in proteorhodopsin-containing *Dokdonia* sp. MED134 (15). Also unlike *Dokdonia*, *V. campbellii* does not exhibit light-responsive expression regulation of central C metabolism genes; however, we did observe a shift toward anaplerotic C fixation and diversion of TCA intermediates toward biosynthesis under C limitation also seen in MED134 (15). Higher expression of phosphoenolpyruvate (PEP) synthase PpsA and PEP carboxykinase PckA under C limitation also suggests that cells could regenerate TCA/glyoxylate cycle intermediates (particularly oxaloacetate) via anaplerotic reactions (15, 43). Acetate may be a substrate for this regeneration: E. coli cells entering stationary phase begin to reuse acetate previously released to the medium for energy and biosynthesis through the glyoxylate shunt (44, 45). C limitation of *V. campbellii* also resulted in higher abundance of several pentose phosphate pathway enzymes, including 6-phosphogluconate dehydrogenase (Gnd), transketolase (Tkt), and ribose 5-phosphate isomerase A (RpiA).

In stationary phase under nitrogen-limiting conditions, *V. campbellii* appeared to adopt a different strategy for regenerating oxaloacetate (OAA) lost to amino acid biosynthesis, upregulating malate oxidoreductase (MaeB) and malate dehydrogenase (Mdh) to form OAA from malate. Two key enzymes of the Entner-Doudoroff (ED) pathway, phosphogluconate dehydratase IlvD and ketohydroxyglutarate aldolase Eda, were also more abundant under N limitation than under C limitation, suggesting increased glycolytic flux through this pathway, which yields only one ATP per glucose as opposed to the 2 ATP per glucose of Embden-Meyerhof-Parnas glycolysis. A shift toward ED glycolysis under N-limiting conditions has been attributed to the lower protein demand of the enzymes of this pathway (46); these experiments, with an excess of available C over N and abundant O_2_ for nonglycolytic ATP production by oxidative phosphorylation, may present favorable conditions for reliance on the ED pathway.

In general, under either C or N limitation, we suggest that *V. campbellii* adjusts expression levels of central carbon metabolism enzymes to enable continued regeneration of NAD(P)H and biosynthetic intermediates, at the expense of glycolytic production of ATP, by shifting metabolic flux away from EMP glycolysis and toward alternative pathways. Under C limitation, these alternatives appear to be the pentose phosphate and C-sparing glyoxylate pathways, as well as anaplerotic C fixation, while under N limitation, the relatively protein-lean Entner-Doudoroff pathway is favored. The limiting nutrient also drives a shift in the mode of ammonium assimilation (Fig. 4): under N limitation, the glutamine synthetase-glutamine:2-oxoglutatate amidotransferase (GS-GOGAT) pathway is more highly expressed, while under C limitation, there is higher expression of glutamate dehydrogenase (GDH), a pattern consistent with observations in E. coli (47). GDH is typically favored under C/energy stress because ATP is not needed, while GS-GOGAT is preferred under N limitation to regulate the glutamine pool (47).

### N-limited stationary-phase protein expression and rapid cultivability loss.

N-limited *V. campbellii* rapidly became uncultivable and mostly nonviable in stationary phase (Fig. 1). Although this stress response is distinct from the VBNC state (which also causes declines in cultivability), proteome analysis of the N-limited culture indicates notable similarities to and some differences from previously observed expression patterns in *Vibrio* induced into the VBNC state by other stressors (Fig. 3). Ribosomal proteins (RplA, -I, -J, -K, -L, -O, -Q, and -Y and RpsF, -H, -M, and -P) maintained abundance in N-limited stationary phase, similar to the VBNC state (48), despite the relatively large proportion of cellular N committed to ribosomes. Cytoplasmic membrane fatty acids and peptidoglycan undergo compositional changes in the VBNC state (28, 49, 50), and we observed proteomic signals of membrane and cell envelope changes as the N-limited cells entered stationary phase, including increased expression of outer membrane lipoprotein carrier protein LolA, isoprenoid biosynthesis proteins IspG and IspF, lipopolysaccharide export system protein LptA, fatty acid acyl carrier protein AcpP, periplasmic stress response proteins SurA and outer membrane protein H precursor Skp, acyl carrier protein IpxA, and TonB-dependent receptors. In Vibrio harveyi, phosphate transporter PstS was downregulated during late stationary phase in a survival experiment, similarly to the case with our N-limited cultures (51). Yet while VBNC cells typically are dwarfed and transition from rod shaped to spherical (28), noncultivable N-limited *V. campbellii* cells retained their rod shape and cultivable C-limited cells became smaller and coccoid (Fig. 1E and F). MreB determines the rod shape of bacteria, and decreased expression would indicate a more spherical shape, as commonly observed in VBNC cells (52), though we observed a decrease in MreB expression under the N-limited condition (49).

Resuscitation from the VBNC state has also been linked to quorum sensing; in V. cholerae and V. vulnificus, quorum-sensing autoinducers have been shown to be molecular signals triggering resuscitation of VBNC cells (53, 54). In *V. campbellii*, we observed higher abundance of autoinducer production protein LuxS under N limitation (Fig. 3), and it is possible that a requirement for sufficient autoinducer production and accumulation under specific (but as yet unidentified) conditions may have contributed to our inability to resuscitate N-starved *V. campbellii*. Altogether, these experiments suggest that N limitation drives rapid viability and cultivability loss in *V. campbellii* but may also inhibit some of the physiological responses associated with the VBNC state.

### C-limited stationary-phase protein expression and stringent response.

C-limited *V. campbellii* cultures lost OD but maintained some degree of cultivability much longer into stationary phase than N-limited cultures. We observed upregulation of a number of proteins associated with phosphate limitation, including PhoB and PstS (Fig. 3), in the C-limited compared to the N-limited stationary phase. However, both media had the same replete concentrations of phosphate, suggesting that these observed expression patterns may in fact be responses to carbon stress, mediated by the stringent response, which is recognized to be involved in dormancy transitions (26). The stringent stress response in many bacteria, including *Vibrio*, is signaled by intracellular accumulation of (p)ppGpp synthesized by RelA and/or SpoT (55), which modulates the stability and activity of a wide array of transcription factors, including RpoS. In E. coli’s stringent response, induction of *phoA* and *pstS* in response to phosphate starvation are dependent on ppGpp synthesized via the *spoT* pathway, not *relA* (56, 57). Under the C-limited conditions, we observed higher overall expression of SpoT (RelA was not detected at the protein level under either the C- or N-limited condition), suggesting that Vibrio campbellii’s C-limited stringent response and generation of (p)ppGpp are mediated by SpoT, which is also necessary for Pho regulon induction during inorganic phosphate starvation in E. coli (58, 59). Again, transcript-level expression and protein-level expression of RpoS (60) (Fig. S4) confirm that both C- and N-limited cultures engage stringent-response transcriptional programs by stationary phase. While the effects of the stringent response in the N-limited cells are less clear, we observed in stationary phase a higher proteomic expression of stringent response protein A (SspA), a stringent response transcription factor which has previously been linked to amino acid starvation, acid resistance, and virulence (61–64). Together, these observations suggest that the stringent responses in Vibrio campbellii to carbon limitation and nitrogen starvation differ and cause distinct downstream effects on gene expression.

Pho regulon genes, including inorganic phosphate-specific transport proteins (PstS and PstB) and phosphate regulon transcriptional regulatory protein PhoB, were all more highly expressed in stationary phase under C than N limitation, despite concentrations of phosphate being replete in both the N- and C-limited media (Fig. 3). “Cross talk” between Pho regulon expression and carbon starvation has been previously observed (65)—possibly since a range of biomolecules contain both phosphorus and carbon, promoting uptake of both when either is limiting. In E. coli, the *ugp* operon, which encodes the glycerol-3-phosphate uptake system, is upregulated by *phoR* and *phoB* under phosphorus starvation and cAMP receptor protein gene *crp* in response to carbon starvation (66, 67). In our experiments, PhoB, glycerol-3-phosphate ABC transporter UgpB, and Crp were all more highly expressed under C limitation, while PhoR was somewhat more highly expressed under N limitation. Additional Pho-regulated genes controlled by carbon sources include those for glycerophosphodiester phosphodiesterase (GlpQ) proteins and 5′-nucleotidases (UshA) (68–71), both of which showed higher expression under C limitation. Interestingly, we also observed higher expression of well-known inorganic phosphate scavenging alkaline phosphatases under C limitation, which has also been seen during P-stimulated anaplerotic C fixation by mesopelagic heterotrophs (72). Phosphorus acquisition did not apparently drive storage in major cellular P reservoirs like Ppk-produced polyphosphate or rRNA (as inferred from ribosomal proteins), which were all more highly expressed under N-limited conditions or not detected. Additional P may be incorporated under C-limiting conditions into ATP via higher expression of phosphotransacetylase (Pta) and acetate kinase (AckA) (73). Our observations are consistent with the hypothesis that the Pho regulon is important in multiple stress and survival responses of vibrios and is not exclusively involved in phosphate starvation (74).

The results of this work show that Vibrio campbellii CAIM 519, despite encoding and expressing proteorhodopsin (PR), did not realize a detectable growth or survival advantage from light-driven metabolism under the carbon or nitrogen limitation conditions tested. While likely an imperfect simulation of a natural *Vibrio* ecological scenario (e.g., particle colonization followed by rapid growth and then substrate exhaustion), these experimental conditions enabled straightforward imposition of defined nutrient limitation while also affording sufficient biomass for direct protein-level quantification of proteorhodopsin. Proteorhodopsin protein was expressed from late exponential through stationary phase, 4.4-fold more highly under C-limiting than N-limiting conditions, but PR expression was not strongly light responsive. PR transcription peaked during the transition from exponential to stationary phase (consistent with regulation by the RpoS stress response sigma factor [13]) but declined during stationary phase while PR protein remained abundant, suggesting that PR transcript abundances may not reliably track protein levels for chronically nutrient-limited cells. Expression of roughly 5,600 copies/cell of PR, even under C-limiting conditions, was not sufficient to measurably affect growth physiology or cellular viability. Overall, only 11 of the 1,933 proteins whose expression we quantified in this nominal photoheterotroph were differentially abundant between light and dark conditions; most of these appear to be related to coping with elevated ROS stress in the light. N and C limitation resulted in broadly different protein expression patterns and survival physiology, with N limitation driving more severe loss of viability and cultivability. Taken together, these results illustrate the challenges in drawing physiological inferences from genome content in aquatic photoheterotrophic microbes. Future developments in molecular physiological techniques (e.g., reducing sample size requirements for proteomic quantification of proteorhodopsin) will be important for studying proteorhodopsin expression and photophysiology at lower cell densities and growth rates more representative of natural planktonic conditions and will help clarify the ecological impact of this widespread but enigmatic protein.

## MATERIALS AND METHODS

### Bacterial growth conditions and sampling.

Vibrio campbellii CAIM 519 (DSM 19270) was cultured in marine broth (Difco) overnight and transferred to 10% marine broth and 90% artificial seawater (ASW) (75, 76) for an additional overnight incubation. Cells were grown in a Percival AR22LC8 incubator at 28°C with continuous illumination (300 μmol of photons m^−2^ s^−1^) and continuous shaking (240 rpm). After reaching optical densities of approximately 0.25, cells were pelleted by centrifugation at 7,197 × *g* for 3 min, washed two times, and resuspended in defined media (Table S1) to a final OD_660_ of approximately 0.4. For gene expression experiments, 100 mL of medium was inoculated with 1.25 mL of starting culture. For smaller-scale growth experiments, 10 mL of medium was inoculated with 0.125 mL of starting culture. The carbon-limited growth medium contained 2.78 mM maltose and 2 mM NH_4_Cl (16.68:1 C/N molar ratio) in ASW. The nitrogen-limited growth medium contained 8.33 mM maltose and 1 mM NH_4_Cl (99.96:1 C/N molar ratio) in ASW. These ratios were selected for equivalent growth yields while clearly limiting growth by their respective nutrients (Table S1; Fig. S1). All experiments were performed in triplicate. The experiments lasted between 172 and 1,637 h, during which the cultures were exposed to either continuous light (300 μmol of photons m^−2^ s^−1^) or continuous dark.

Samples were collected every 1.5 h for the first 15 h and every 24 h thereafter. Proteomic samples (4.5-mL volume) and reverse transcription (RT)-qPCR samples (1.5-mL volume) were pelleted by centrifugation (7,197 × *g* for 3 min and 11,000 × *g* for 1.5 min, respectively), supernatant was discarded, and samples were flash frozen on liquid nitrogen and stored at −80°C. CFU were determined by serial dilution and spot plating on marine agar. Flow cytometry and microscopy samples (1-mL volume) were fixed with 10 μL of 25% glutaraldehyde in the dark for 10 min and flash frozen for −80°C storage.

### Cell lysis, peptide fraction preparation, and isotope labeling.

Membrane protein enrichment was performed with an adapted carbonate extraction protocol (77, 78). Cell pellets were resuspended in 333 μL of wash solution (50 mM Tris-HCl [pH 7.5]), lysed with high-power sonication (QSonica Q500; 15 min, 1-s pulse/1-s pause, 85% amplitude), and centrifuged (2,500 × *g*, 8 min) to pellet unlysed debris. Supernatant was added to 830 μL of 100 mM sodium carbonate in a polypropylene microcentrifuge tube and shaken at 4°C for 1 h. Membranes were pelleted in an Optima MAX-XP Beckman Coulter ultracentrifuge (115,000 × *g*, 1 h). Supernatant was drawn off as the “cytosolic” fraction.

Cytosolic fraction samples were diluted 1:1 in exchange buffer (8 M urea, 0.2% [wt/vol] deoxycholate, 1 M ammonium bicarbonate) plus 20 mM dithiothreitol (DTT). Membrane pellets were disrupted with high-power sonication (QSonica Q500; 10 min, 1-s pulse/1-s pause, 85% amplitude) in 500 μL of LDS buffer (137 mM Tris HCl, 140 mM Tris Base, 73 mM LDS, 513 μM EDTA, 1.08 M glycerol) plus 20 mM DTT. Membrane fraction samples were incubated at 95°C (20 min) and then at 37°C (30 min) before both fractions’ cysteine thiols were alkylated with 60 mM iodoacetamide (1 h, dark). Protein extracts were purified using an enhanced FASP (eFASP) protocol (79); membrane fraction proteins were digested first with 2 μg of mass spectrometry (MS)-grade chymotrypsin (room temperature, overnight) and then 2 μg of trypsin (room temperature, an additional overnight), while cytosolic fraction proteins were digested only with trypsin (room temperature, overnight). Peptides were eluted and dried by vacuum centrifugation. Peptide samples were resuspended in 2% acetonitrile plus 0.1% formic acid and divided 2/3 by volume for quantitative diDO-IPTL (dimethylation-deuteration and oxygen-exchange isobaric peptide terminal labeling) and 1/3 by volume for PR quantification using labeled standard peptides. Quantitative diDO-IPTL subsamples were dried again by vacuum centrifugation in preparation for isotopic labeling.

Cytosolic and membrane fraction peptide samples were each isotopically labeled for quantitative analysis by dimethylation at N termini with d2-formaldehyde for membrane fractions (CD_2_O, 98 atom% D; CDN Isotopes) or unlabeled CH_2_O (Thermo Pierce) for cytosolic fractions and by enzyme-catalyzed oxygen exchange at C termini with ^16^O-water for membrane fractions (99.99 atom% ^16^O; Sigma) or with ^18^O-water (98.5 atom% ^18^O; Rotem) for cytosolic fractions, following the diDO-IPTL methodology (19). For membrane fraction samples, C-terminal O isotope exchange was performed first with 2 μg of chymotrypsin (room temperature, overnight) and then additionally with 2 μg of trypsin (room temperature, additional overnight).

### Standards for quantitative proteomics.

To generate internal standards for whole-proteome quantification by diDO-IPTL, Vibrio campbellii CAIM 519 was grown in three media (C-limited and N-limited media and 10% marine broth as described above; 100-mL cultures) in both continuous light and dark. Twenty milliliters was collected from C-limited and N-limited cultures at 10, 15, and 99.5 h and at 4, 7, and 100 h from 10% marine broth cultures, so that the standard would represent exponential, transitional, and stationary growth phases. Cytosolic and membrane peptide fractions from the standard were prepared and diDO-IPTL labeled conversely to the samples described above (i.e., CD_2_O/H_2_^16^O for cytosolic fractions and CH_2_O/H_2_^18^O for membrane fractions). Because of the large volume of cytosolic fraction samples, samples were concentrated in a 50-mL Amicon centrifugal filter unit (30-kDa cutoff) between the dilution in 1:1 exchange buffer step and eFASP. Labeled peptides from all 18 standard aliquots were combined to produce a pooled internal standard for each of the membrane and cytosolic fractions. For liquid chromatography (LC)-MS analysis, 3 μL of labeled internal standard was mixed with 5 μL of labeled sample peptides.

Because proteorhodopsin could not be consistently detected in our membrane fraction samples following IPTL labeling, we adopted a synthetic-standard approach for PR quantification. Two *V. campbellii* PR peptides (LWETQGVAK and NLADVVNK) that were consistently detected in unlabeled membrane fractions were selected as quantification targets and a stock solution of synthetic peptides containing ^13^C_6_,^15^N_2_-lysine (New England Peptide) prepared at 0.5 pmol/μL. A 0.75-μL volume of standard peptide stock was added to 7.25 μL of unlabeled membrane fraction sample for LC-MS analysis.

### Proteomic LC-MS.

For LC-MS analysis, 6 μL of peptide sample/standard mix was injected onto a trapping column (OptiPak C_18_; Optimize Technologies) and separated on a monolithic capillary C_18_ column (GL Sciences Monocap Ultra, 100 μm [internal diameter] by 200 cm [length]) using a water-acetonitrile plus 0.1% formic acid gradient (2 to 50% AcN over 180 min) at 360 nL/min using a Dionex Ultimate 3000 LC system with nanoelectrospray ionization (Proxeon Nanospray Flex). Mass spectra were collected on an Orbitrap Elite mass spectrometer (Thermo Scientific) operating in a data-dependent acquisition (DDA) mode, with one high-resolution (120,000 *m*/Δ*m*) MS1 parent ion full scan triggering Rapid-mode 15 MS2 CID fragment ion scans of intensity-selected precursors. Only multiply charged parent ions were selected for fragmentation, and dynamic exclusion was enabled with a duration of 25 s and an exclusion window of ±15 ppm.

### Quantitative proteomics data analysis.

diDO-IPTL mass spectra were matched to the *V. campbellii* CAIM 519 translated genome (80) and isotopologue abundance ratios were quantified using MorpheusFromAnotherPlace (MFAP) (81). Spectrum-level FDR for the diDO-IPTL data sets was controlled using *q* values to <0.1%. A total of 1,933 proteins were quantified in at least one sample; 266 proteins were quantified only in the membrane fraction and 958 proteins only in the cytosolic fraction. For the 709 proteins quantified in both cytosolic and membrane fractions, expression results from the fraction with the greater number of quantified spectra were used (493 proteins designated to the cytosolic fraction and 216 proteins designated to the membrane fraction). Proteomic cultures were sampled every 1.5 h between hours 7.5 to 15 and then again at hours 27.5, 51.5, and 75.5; the first 3 time points were designated exponential phase, the next 4 time points transition phase, and the final 2 time points stationary phase. diDO-IPTL data are normalized by setting the median of the set of all log_2_-transformed protein abundance ratios in each sample to zero; this normalizes for small in variations in sample size, extraction/digestion yield, etc., between samples and produces abundance ratios on a per-amount-total-protein basis. Data from biological triplicates were consolidated for each protein by taking the mean log_2_ abundance across replicates and calculating the associated error as [(σ_x_)^2^ + (μ_SE_)^2^]^1/2^, where σ_x_ is the standard deviation of normalized log_2_ expression values between replicates and μ_SE_ is the mean of the standard error of normalized log_2_ expression values (calculated by MFAP) across replicates. Only peptides represented by two or more IPTL spectra were retained for further analysis. Statistically significant differential expression between growth conditions was determined using limma pairwise comparison contrast matrices, pooling samples within a growth phase (82). PR quantification was performed by manual MS1 peak area integration in Xcalibur (Thermo Scientific) of the two target peptides from the samples and the isotopically labeled standards; errors represent the standard deviations between biological triplicates of sample/standard ratios averaged for the two quantified peptides.

### RT-qPCR.

Primers were designed for four different genes: the proteorhodopsin gene, the beta-carotene 15,15-monooxygenase gene (*blh*), *rpoS*, and *recA* (Table S4). Primer pair efficiencies were confirmed using PowerUp SYBR green master mix (Thermo Scientific) (83). RNA was extracted using the *Quick*-RNA fungal/bacterial miniprep kit (Zymo Research) and purified by DNase treatment (TURBO DNA-free kit; Invitrogen). One-step qPCRs (iTaq universal SYBR green one-step kit; Bio-Rad) were performed on a CFX96 Touch real-time PCR system (Bio-Rad); *recA* was analyzed as a control gene, and errors represent the standard deviation of biological triplicates.

10.1128/mSystems.01263-21.10 TABLE S4 Primer sequences used for transcript-level expression profiles of the proteorhodopsin gene, *blh*, *rpoS*, and *recA*. *recA* was used for normalization. Download Table S4, PDF file, 0.2 MB. Copyright © 2022 Gallagher and Waldbauer. 2022 Gallagher and Waldbauer. https://creativecommons.org/licenses/by/4.0/ This content is distributed under the terms of the Creative Commons Attribution 4.0 International license.

### Microscopy, flow cytometry, and ATP assays.

Wet-mount, fixed cells were imaged using red autofluorescence at a magnification of ×100 on an Olympus IX81 inverted wide-field microscope. Cell counts were done by staining with SYBR gold (Thermo), and viability was assayed using the LIVE/DEAD BacLight bacterial viability kit (Thermo), with flow cytometry performed on a CytoFLEX S (Beckman Coulter). Cellular ATP was assayed with the Bac-Titer Glo kit (Promega); heat shock lysis was performed at 90°C for 5 min.

### Data availability.

Proteomic mass spectral data are available via the MassIVE repository (massive.ucsd.edu) under accession number MSV000087100.
