# Supplementary figures and images for: Proteome Expression and Survival Strategies of a Proteorhodopsin-Containing Vibrio Strain under Carbon and Nitrogen Limitation
Source: mSystems. 2022 Apr 6;7(2):e01263-21. doi: 10.1128/msystems.01263-21 (PMC9040609; doi:10.1128/msystems.01263-21)

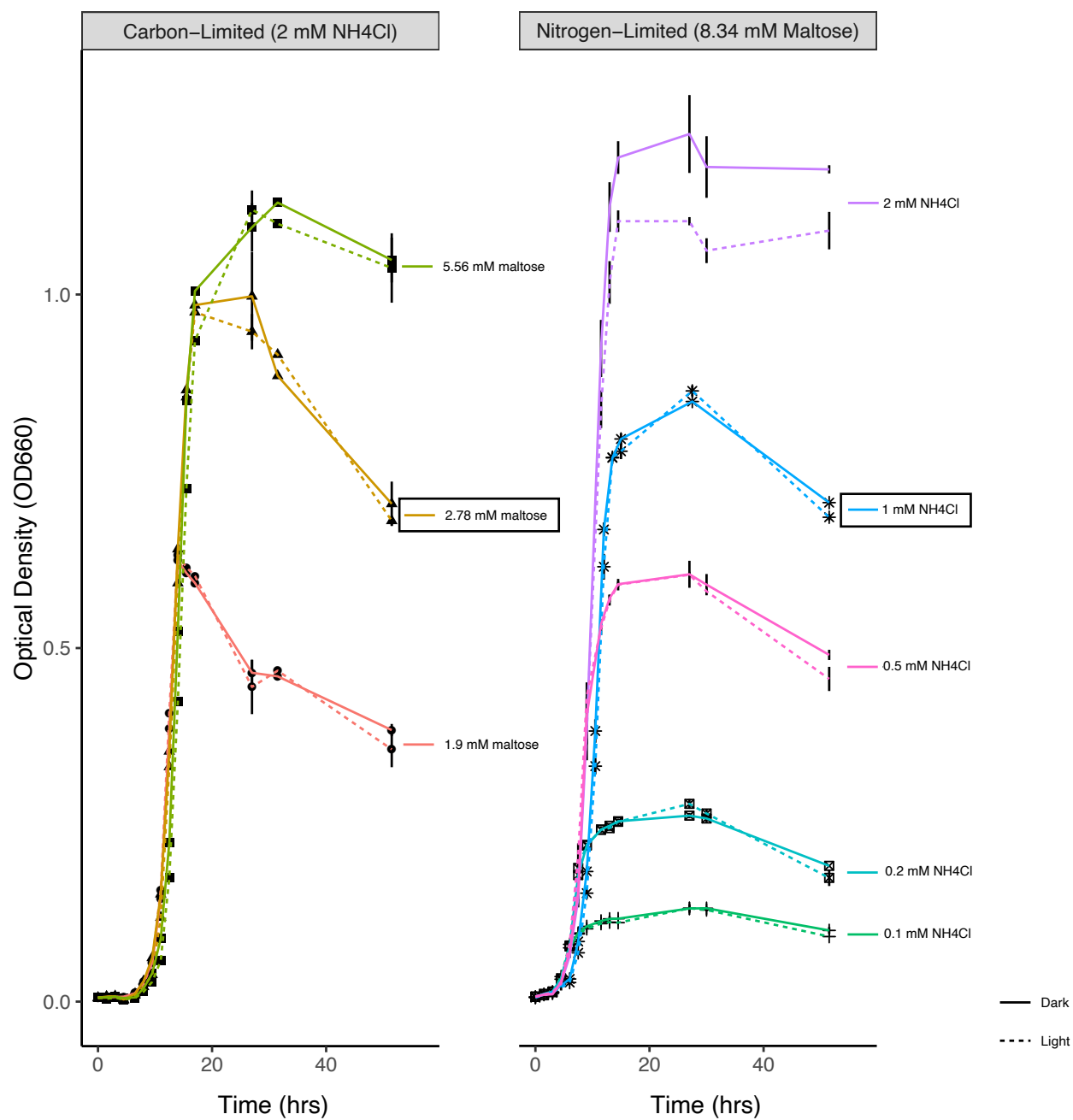

Supplement: FIG S1 [file msystems.01263-21-sf001.pdf]

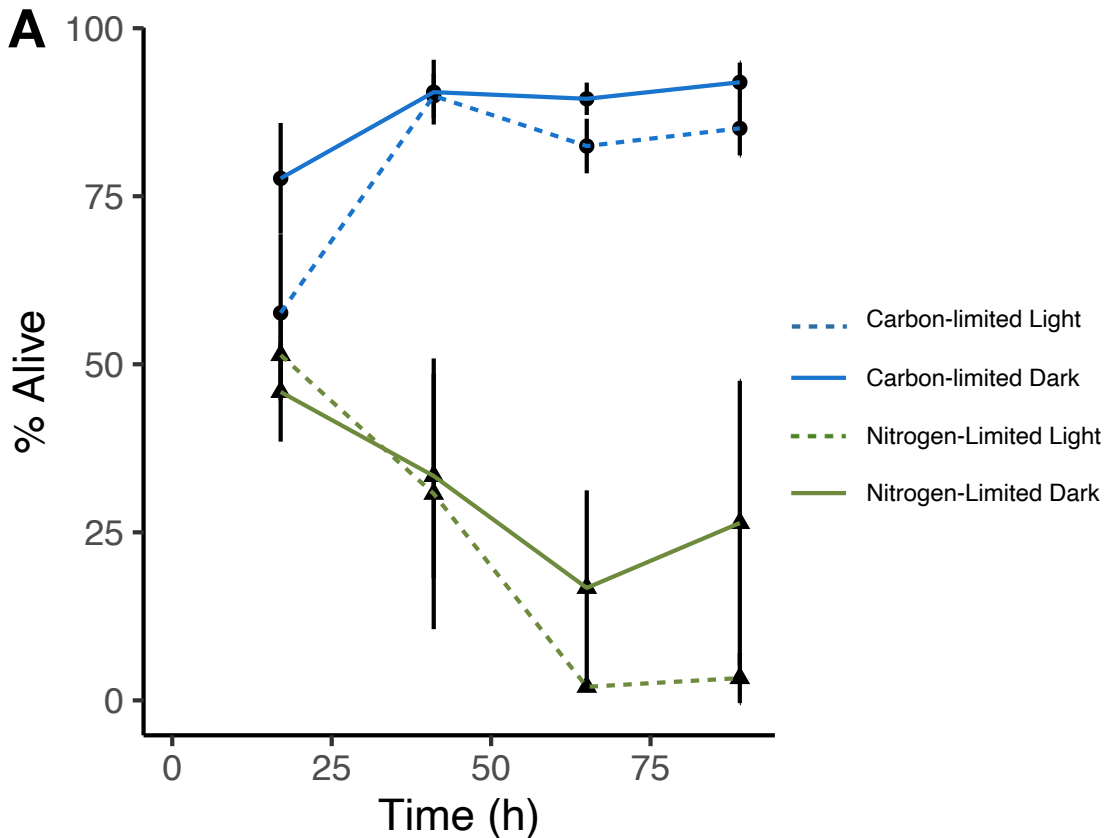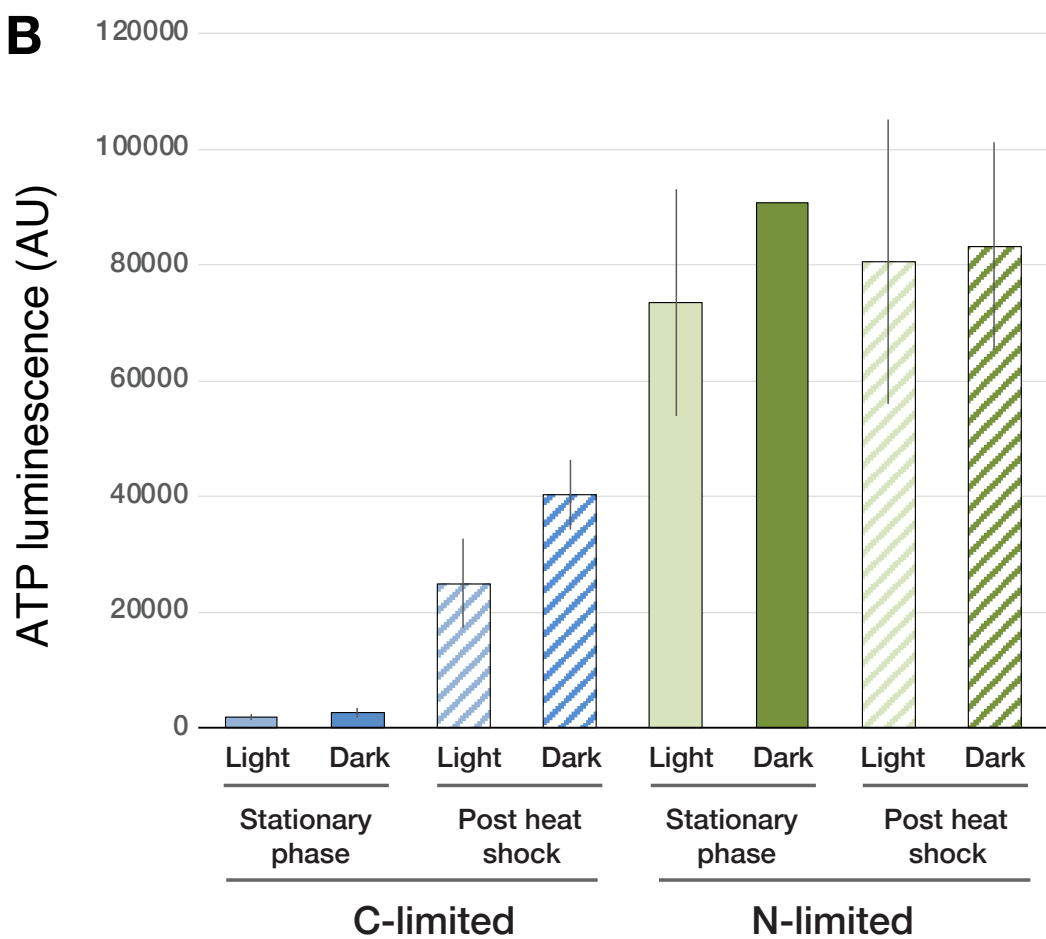

Supplement: FIG S2 [file msystems.01263-21-sf002.pdf]

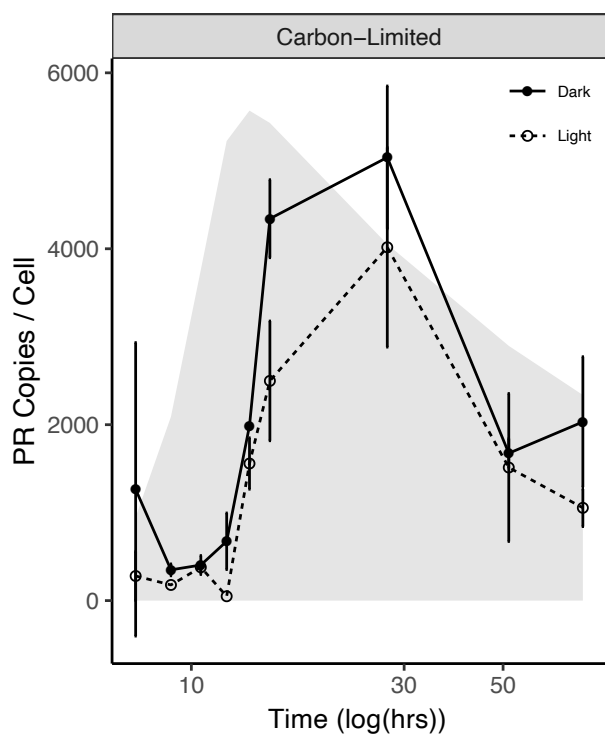

Supplement: FIG S3 [file msystems.01263-21-sf003.pdf]

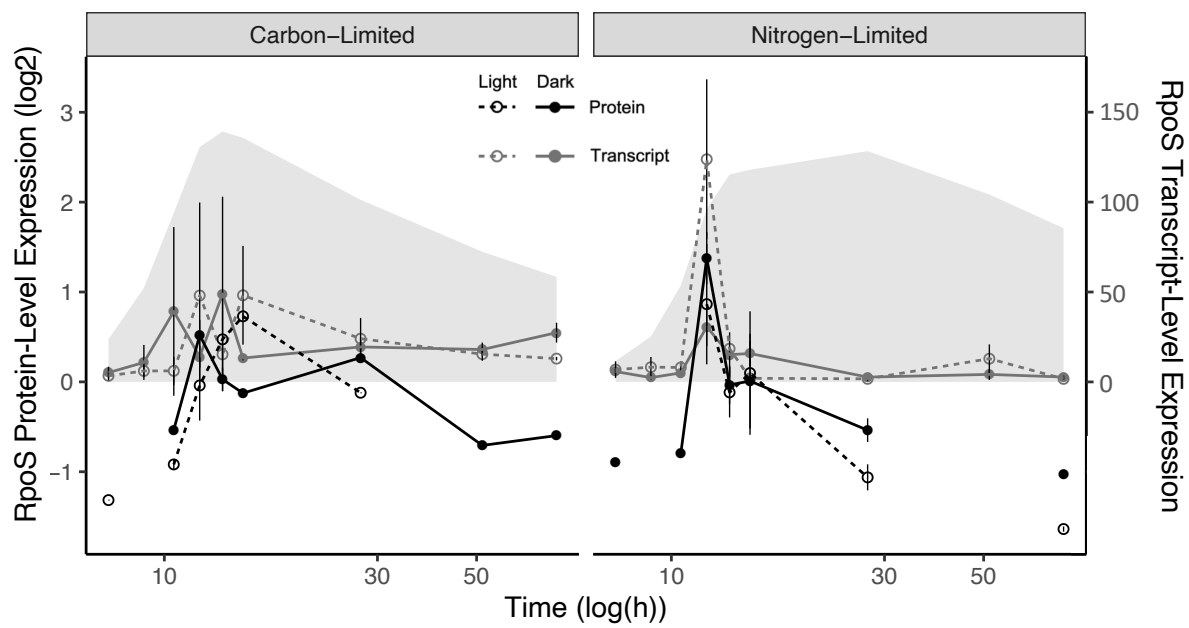

Supplement: FIG S4 [file msystems.01263-21-sf004.pdf]

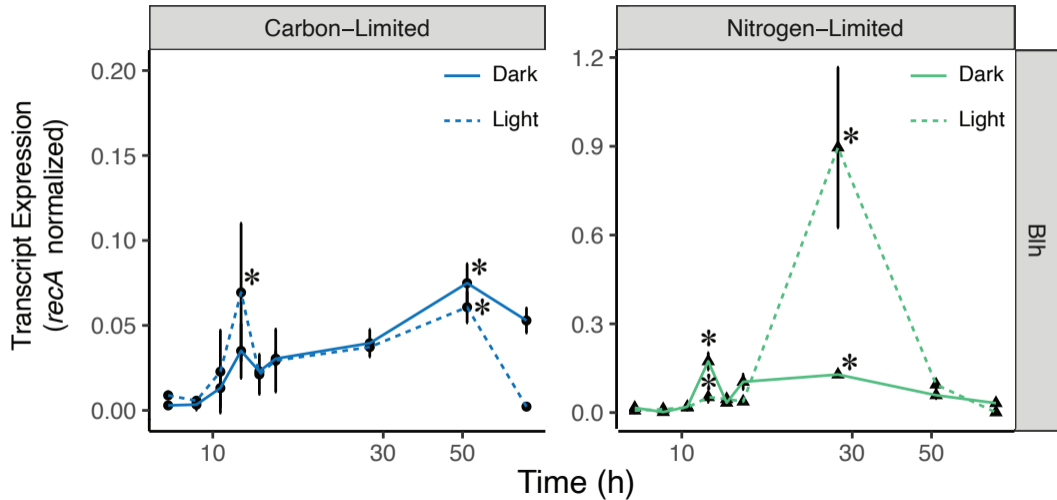

Supplement: FIG S5 [file msystems.01263-21-sf005.pdf]

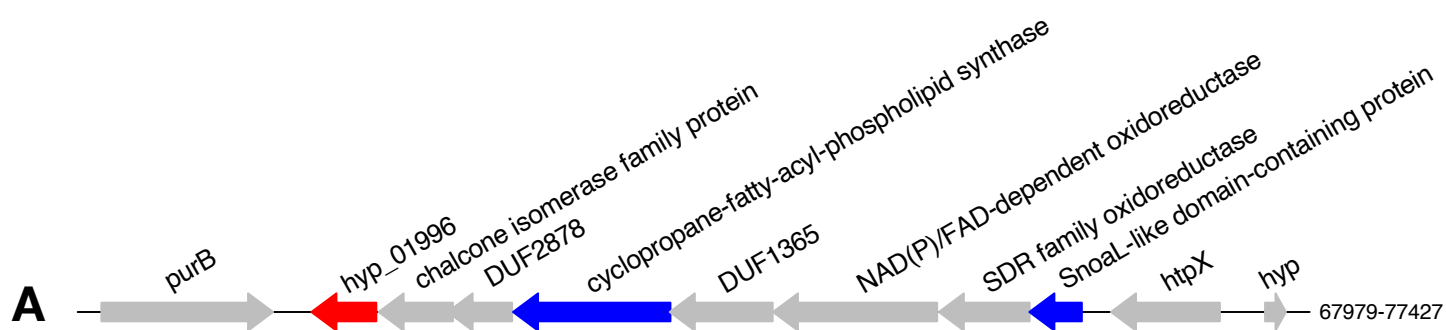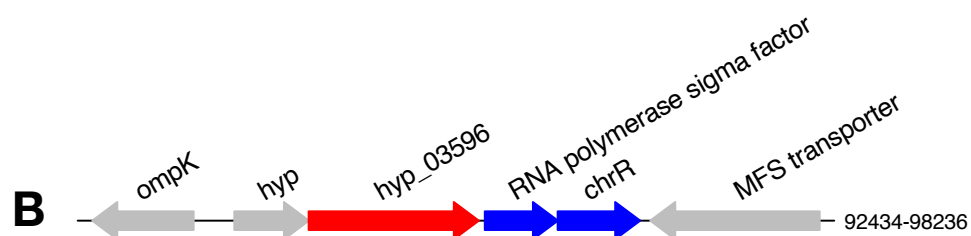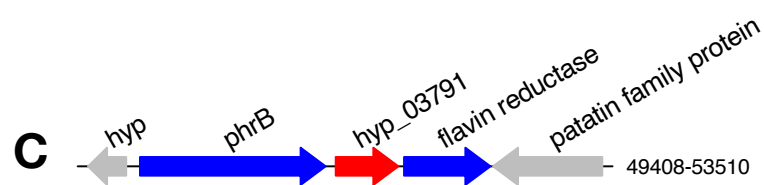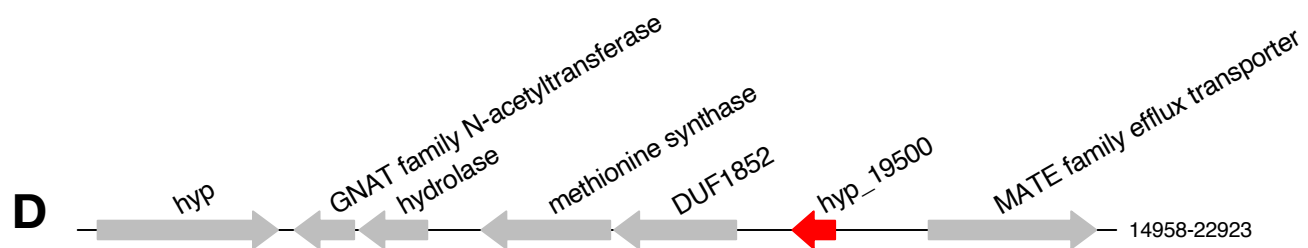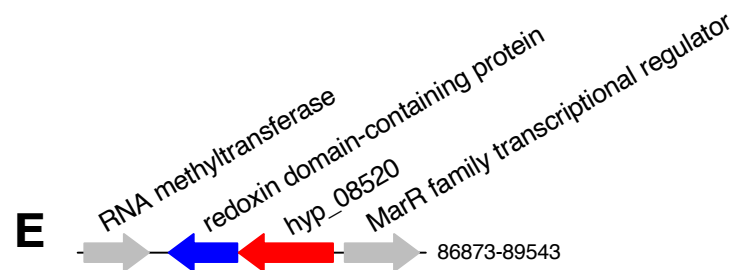

1 kb

Supplement: FIG S6 [file msystems.01263-21-sf006.pdf]
