# Supplementary material for: Proteome Expression and Survival Strategies of a Proteorhodopsin-Containing Vibrio Strain under Carbon and Nitrogen Limitation
Source: mSystems. 2022 Apr 6;7(2):e01263-21. doi: 10.1128/msystems.01263-21 (PMC9040609; doi:10.1128/msystems.01263-21)
Supplement: TABLE S1 [file msystems.01263-21-st001.pdf]

Component	C-limited ASW	N-limited ASW
NaCl	428 mM	
$MgCl_2 \cdot 6H_2O$	9.8 mM	
KCl	6.7 mM	
$MgSO_4 \cdot 7H_2O$	14.2 mM	
$CaCl_2 \cdot H_2O$	3.4 mM	
Tris	9.1 mM	
Maltose	2.78 mM	8.34 mM
Adjusted to pH 8.1		
$FeCl_3 \cdot 6H_2O$	11 μM	
$Na_2EDTA \cdot 2H_2O$	1.3 μM	
H_3BO_3	46.278 μM	
$MnCl_2 \cdot 4H_2O$	9.15 μM	
$ZnSO_4 \cdot 7H_2O$	0.772 μM	
$CuSO_4 \cdot 6H_2O$	0.032 μM	
$CoCl_2 \cdot 6H_2O$	0.025 μM	
$Na_2MoO_4 \cdot 2H_2O$	1.616 μM	
$NaH_2PO_4 \cdot H_2O$	0.13 mM	
$NaHCO_3$	5.9 mM	
NH_4Cl	2 mM	1 mM
