# Supplementary material for: Proteome Expression and Survival Strategies of a Proteorhodopsin-Containing Vibrio Strain under Carbon and Nitrogen Limitation
Source: mSystems. 2022 Apr 6;7(2):e01263-21. doi: 10.1128/msystems.01263-21 (PMC9040609; doi:10.1128/msystems.01263-21)
Supplement: TABLE S2 [file msystems.01263-21-st002.pdf]

Nutrient condition	Light/Dark	Time (h)	n Cells measured	Mean max Feret diam (μm)	Max Feret diam SD	Mean min Feret diam (μm)	Min Feret diam SD	Aspect ratio (max:min diameters)
Carbon-limited	D	10.5	37	3.49	1.03	2.04	0.43	1.71
		17.5	73	5.07	3.51	1.77	0.46	2.86
		148	127	1.78	0.50	1.42	0.35	1.25
	L	10.5	96	3.30	1.45	2.06	0.54	1.60
		17.5	69	5.78	4.02	2.24	0.55	2.58
		148	124	1.79	0.88	1.38	0.33	1.30
Nitrogen-limited	D	17.5	38	3.13	1.29	1.82	0.49	1.72
		27	46	5.20	3.61	2.22	0.71	2.35
		148	42	6.24	4.30	2.45	0.71	2.55
	L	17.5	38	3.98	2.49	1.99	0.63	2.00
		27	47	5.48	4.64	2.09	0.48	2.63
		148	42	4.97	2.54	2.17	0.51	2.29
