# Supplementary material for: Proteome Expression and Survival Strategies of a Proteorhodopsin-Containing Vibrio Strain under Carbon and Nitrogen Limitation
Source: mSystems. 2022 Apr 6;7(2):e01263-21. doi: 10.1128/msystems.01263-21 (PMC9040609; doi:10.1128/msystems.01263-21)
Supplement: TABLE S4 [file msystems.01263-21-st004.pdf]

Name	Sequence	Length (bp)	Tm	GC%	Pdt Length
Vc_PR_1576282_F	GGGATGTTTGGGTCGCTACA	20	60.04	55	133
Vc_PR_1576414_R	AATATACCAGCGGAGGCTGC	20	59.96	55	133
Vc_blh_1581413_F	GCTACTCAATTGGGAGCGGT	20	60.11	55	84
Vc_blh_1581496_R	GGGGGTGAGGTCACTAAACG	20	60.04	60	84
Vc_rpoS_2774862_F	CTCGTACAATCCGTCTGCCA	20	59.83	55	77
Vc_rpoS_2774786_R	CTGAGAAAGTTCACGCGCAG	20	59.84	55	77
Vc_recA_2770048_F	AGGTGACAAGATCGGCCAAG	20	60.04	55	124
Vc_recA_2769925_R	TGCTTCAGGTAGTGCTGGTG	20	59.96	55	124
